# The Spanish Language as a Cultural and Touristic Resource for the Chinese Market to Develop Quality Education

**DOI:** 10.3389/fpsyg.2021.815350

**Published:** 2022-02-01

**Authors:** Blanca García-Henche, Ming Yang

**Affiliations:** ^1^Economics and Business Management Department, Faculty of Economics, Business and Tourism, Universidad de Alcalá, Alcalá de Henares, Spain; ^2^Department of Spanish, School of European and Latin American Studies, Shanghai International Studies University, Shanghai, China

**Keywords:** language tourism, Spanish learning, cultural tourism, Chinese tourism market, international cooperation

## Abstract

Since 1952, Spanish has been included as a Degree in the Foreign Language Studies in the higher education system of China. The number of Spanish students has gradually increased and, until March 2020, with 6 Universities recently approved by the Chinese Ministry of Education (MOE), there are 102 Chinese universities that teach Spanish as a university degree. In 2017, the MOE of the People's Republic of China (PRC) published the Curriculum Plan in the Higher Secondary Schools, which incorporated the teaching of Spanish as a teaching option along with other languages, such as English, Japanese, Russian, German, and French. In the following year, during the Chinese President's state visit to Spain, a cooperation agreement was signed between the Royal Spanish Academy (abbreviated as RAE for its Spanish name) and SISU, under which a RAE-SISU Joint Research Centre was established. This study aims to publicize the situation of Spanish teaching in China, showing how a language can become a cultural and language tourism resource for Spain. An empirical study of student profiles was carried out at one of the most prestigious universities with reference to Spanish teaching in China, SISU. The data were obtained through primary sources employing a survey sent to the Chinese students studying their bachelor's degree of Spanish Philology, obtaining a sample of 159 students. The results revealed information on the studies carried out, also reviewing the most popular universities among the Chinese students in Spain, their stay period, and the destination image that the students have. The data will serve to show the opportunities for Spain as a destination for language tourism for the Chinese market. One of the implications of this study would be that it helps to explain how Spain can design a product for a niche market that demands Spain as a destination for language and cultural tourism.

## Introduction

Traveling to other countries to learn a language or to improve individuals' language skills has become a worldwide trend in the last decades for a broad spectrum of population segments (Iglesias, 2018). This study intends to give an account of the possibilities of the Spanish tourism sector in the face of a new niche of the tourism market that supposes the linguistic tourism from China to Spain.

In Spain, the teaching of Spanish to foreigners has traditionally been considered a mere educational activity. At present, it has come to be interpreted as a cultural tourism subsector, and in the most recent promotions, specific actions have been included to promote language tourism, as have been done by the United Kingdom, France, Germany, or the United States, with languages of obvious international relevance.

In the strategic plans of language tourism in Spain, prepared by the Spanish Federation of Schools of Spanish as a Foreign Language (FEDELE), it is stated that the main tourism agents have long been promoting language tourism as a subsegment of cultural tourism of special interest (FEDELE Federación de Escuelas de Enseñanza de Español para Extranjeros, 2009, 2016, 2018). On the other hand, in the recent years, the tourist promotions of the Tourism Institute of Spain (Turespaña) already have concrete actions for the development and promotion of language tourism in our country, especially in the Asian countries, such as Japan, Korea, or China with an important growth of Spanish students who represent a key target market for language tourism.

Although the economic boom has turned China into a voracious market where we can expand our language and culture, Spanish is still a *xiao yu zhong*, that is, a minority language, in the Asian giant. Even so, there are many efforts that have been carried out from both countries to promote the teaching of Spanish as a foreign language. On the other hand, the increasingly stable relations between China and Latin America, through investment and trade, bring as a result, the great need to have highly qualified bilingual personnel both in the Chinese and the Spanish.

According to Lu (2012), one of the most renowned professors of Hispanic Philology, Dean of the Faculty of Western Philology of Shanghai International Studies University (2005–2014), the national coordinator of Spanish Teaching in China (1997–2020) and the president of the Asian Association of Hispanics (2010–2013), despite a muted growth, there's a great demand for Spanish in China, where Hispanic Philology is taught in many universities as the most chosen career. Lu Jing Sheng (2012) adds that the students with the highest grade of the *gao kao* (university entrance exam) in Shanghai and Beijing, in the recent years, have decided to study Hispanic Philology; so this would be a testimony to the increasing scope of Spanish in this society and that there are people trained in China who are aware of the importance of Spanish in the world.

The history of foreign language teaching in China has always been related to the principle of safeguarding and ensuring the national interests since the foundation of the People's Republic of China (PRC). According to the list of regular institutions of higher learning, till 2021, there are 23 universities which have literally “foreign languages” in their official name, as specialized universities devoted explicitly to teach foreign languages and cultures in the higher education phase. Among them, there are four institutions with the longest history, namely the Beijing Foreign Studies University (BFSU), SISU, the Sichuan International Studies University, and the Xi'an International Studies University, whose foundation dated back respectively to 1941, 1949, 1950, and 1951. Many investigators (Qu, 2019; Shen, 2019; Shu, 2019, 2021; Wen, 2019) have published articles on 70 years of foreign language education planning of China and its foreign language teaching and research on the 70th Anniversary of PRC. As Qu (2019) suggested, “foreign language teaching” refers to the teaching of foreign languages in the universities as a degree study, based on the blueprint of English teaching. Wen (2019) classified the history of foreign language teaching in China into four periods: lean to Russian side, with English oppressed (1950s); everyone learns English (1960s−1980s); English heat cools down (2000s), and Golden Age for multilingualism (2010s). As Qu (2019) mentioned in his research, the Cultural Revolution, which ended in 1976, was a turning point for the foreign language teaching in China.

Shen (2019) set two paradigms, the instrumental paradigm and the cultural paradigm, for foreign language teaching and discussed its evolution. The categorization of the two paradigms is quite similar to the distinction of sentimental and instrumental motivations (Cooper, 1989) or to the division of two types of language planning, the instrumental and socio-linguistic (Kirkwood, 1990). Since World War II, in order to solve the communication problems, all the emerging countries of the world have established the value of language as a tool in language planning, to obtain social resources. The instrumentalism has presented the leading role of English in national foreign language education planning. Regarding Globalization and linguistic paradoxes in Asian Countries, Tsui (2008) suggested that many Asian countries, such as China, Japan, South Korea, and Malaysia, have established relative policies to ensure English literacy skills of their citizens.

According to the World Bank, since China began to open up and reform its economy in 1978, GDP growth has averaged almost 10% a year, and more than 800 million people have been lifted out of poverty. As the world's second largest economy next to the United States, China, from being a local country, has turned into a regional and international country. Therefore, its instrumental paradigm for foreign language teaching has been suffering changes, gradually and accumulatively. Earlier, in the limited foreign socio-linguistic context, a foreign language was a key or a tool to learn advanced foreign science and technology, their culture, and their high-end administration and management experience, and English has been the most learned language, as *lingua franca*, since 1960s. Nowadays, China is facing the world and committed to the internationalization of various foreign language service needs, involving different fields and practitioners in all walks of life. That is to say, China is pursuing a multipronged strategy toward global governance, for which the demand for foreign languages will be more extensive and the linguistic status of Belt and Road countries will gradually become more relevant. This is the socio-cultural context of the Golden Age of multilingualism.

Since 1952, Spanish has been included as a degree in the foreign language study in the higher education system of China. The number of students learning Spanish has been increasing gradually and, until March 2020, with the six universities recently approved by the Chinese Ministry of Education (MOE), there are 102 Chinese universities that teach Spanish as a university degree. In 2017, the MOE of the People's Republic of China published the Curriculum Plan in Higher Secondary Schools, which incorporated the teaching of Spanish as a teaching option along with other languages, such as English, Japanese, Russian, German, and French. In the following year, during the Chinese President's State visit to Spain and his meeting with his Spanish counterpart, between the Royal Spanish Academy (abbreviated as RAE for its Spanish name) and SISU, a cooperation agreement was signed, under which a Joint Research Centre between RAE and SISU was established.

The moral of the Chinese idiom, “build a cart behind closed doors” is that when one sets out to accomplish something, especially something one has never done before, it is vitally important to first learn the correct method before acting. It is even harder to think a foreign language teaching plan without any Study Abroad program. Authors like Lafford and Isabelli (2019) suggested that one of the important themes in the Study Abroad programs is to develop the intercultural competence, which is defined as “the set of cognitive, affective and behavioral skills and characteristics that support effective and appropriate interaction in different cultural contexts.” With the possibilities for the cultivation of intercultural competence in Spanish-speaking countries, Chinese students may enhance their research capacity in linguistics and literature courses approached with Spanish as L1. Study Abroad programs seem to be the missing puzzle to complete the Spanish teaching as a degree study in China.

Chinese students who study Spanish represent an opportunity for language tourism in Spain, since they know the culture and the language, and they travel to Spain seeking to relate to the language and culture in which they have already been trained. Thus, language tourism is based on the teaching of Spanish in the country of origin but also refers to the teaching of Spanish in Spain as a primary service to which we must add all the complementary services that students demand (plane tickets, accommodation, travel, shopping, visits to museums, etc.).

The Spanish language, thus, becomes an important tourist resource and is positioned in a privileged way when it comes to being studied and practiced by students from the five continents, assuming a tourist product that achieves the diversification and deseasonalization of tourism in Spain as a tourist destination.

The prosperity and stability of our future are inseparable from that of the developing and the least-developed countries. Economic collaboration among different countries is critically important in the transformation of the economic-growth model. Developed countries have made great advances in fields, such as education; these advances can be used to resolve difficulties more effectively. Sharing of these advances among different regions is conducive to improving the ability to cope with the various challenges on the sustainable development goals (SDGs).

In addition, culture, as the combination of knowledge, beliefs, institutions, and customs, profoundly affects human behavior and is the key factor to determining whether sustainable development can be achieved (Global Sustainable Development Report, 2019). Countries and regions comprise diverse cultures; so resolving conflicts among different cultures is critical to achieving policy coherence that promotes sustainable development. Hence, based on respect to cultural differences, we should work together to build a global sustainable culture to encourage joint action among all countries and the study of the language and culture of a country is a great step to generate knowledge and respect for another culture. Therefore, it is considered that the learning of a language, collaboration between educational institutions, and language tourism are key axes for the achievement of the SDGs, especially to Develop Quality Education (SDG4), because education enables upward socioeconomic mobility and is a key to escaping poverty, and International Cooperation (SDG17) as SDGs.

Accordingly, this study is organized as follows: First, the study presents a review of the literature on language tourism. Then, it precisely describes the history of foreign language teaching in China, of Spanish teaching in China, and of Spanish teaching in SISU. It then analyses Spain as a destination for language tourism in China. Finally, the study discusses the results describing the profiles of Spanish students at SISU, the studies carried out in Spain, and the image of Spain as a tourist destination, to show the business opportunities represented by the Asian market niche in language tourism. This study concludes with the theoretical and practical implications and suggestions for future research directions.

## Conceptual Framework

### Research Framework

For decades, tourism has experienced continuous growth and deep diversification, becoming one of the fastest growing economic sectors in the world (Brida et al., 2011). Spanish is a tourist resource with enormous potential, whose *raison d'ies* lies in its growing socioeconomic importance in today's world (Piedrola et al., 2017).

Throughout the world, people travel abroad to learn a foreign language in different ways, and the consequences of such experience have been studied quite extensively since the late 1960s, although research has referred to a limited range of Study Abroad contexts and target languages (Churchill and DuFon, 2006).

It is interesting to note the perspective Thurlow and Jaworski (2011) had on language tourism, according to which, tourist modality leads to the “commodification” and “recontextualization” of language. For them, language becomes a commodity in language tourism trips and adds authenticity to cultural visits, in which language is always a key asset.

According to Alonso and Gutiérrez (2007), the consumption of natural resources implies a process of depletion. On the contrary, the language, considered to be good, is not worn out or exhausted but is also enriched. Its value increases as its consumption expands (Jiménez, 2006; Jiménez-Redondo, 2006).

Language tourism is a very attractive form of tourism due to the growth forecasts in our country, but the knowledge of its market is still scarce. Its studies have only been followed since the year 2001. From this, it is clear, first, the economic importance of language tourism was due to extended stays and, second, the varied offer of educational establishments was composed of universities, private centers, and official schools (Pardo, 2011).

Castillo et al. (2018) highlighted in their study the importance of the role of the international university students as an engine for the promotion of the development of international educational tourism as a modality within language tourism. They highlighted the importance of international educational tourism to promote the diversification of a country's tourism product and, in this way, contribute to its economic growth.

In the bibliography on language tourism, we can find academic research, with scientific methodologies, based on the motivations and preferences of visitors. In the case of Spain, there are different studies related to language tourism. This is the case of the study by Castillo et al. (2018), which was carried out with the purpose of analyzing the reasons why international university students choose Córdoba (Spain) as their destination city. This way, from the data of the said study, improvements can be introduced in the offers with the desire to reinforce the image of Spain, worldwide, to attract more international students.

Rodríguez et al. (2010, 2013) had analyzed the experience of European students in the Spanish universities for the period, 2000–2010, taking into account the three dimensions of sustainable development: economic, environmental, and social. With the data of their study, they justified that academic tourism, due to its characteristics, is a more sustainable type of tourism than conventional tourism, as it has a relatively greater economic impact than the conventional tourism (Ceballos et al., 2020). As a result, the negative effects of academic tourism on the environment are not significant and it is beneficial to future generations, as it contributes to the increasing knowledge and interrelationship among students from different countries and cultures.

All Spanish-speaking countries have Spanish as their official language and constitute the greatest demographic wealth of our language, not only for the number of speakers but also for its economic, cultural, and social attractiveness as a market with a strong and growing demand for consumption. Therefore, there are numerous studies on nautical tourism related to Spain and Latin America which aim to influence tourism and cultural policies, due to the undoubted advantages offered by nautical tourism and all its activities integrated into the receiving community (Huete, 2008; Tamames, 2009; Soto, 2012; Clark, 2014; Iglesias, 2016, 2018; Ullauri et al., 2017).

Although there are no recent official data from Turespaña, according to the data provided by the Instituto Cervantes (2019) the annual number of language tourists is estimated to be 2,000,000, of which, 2,73,000 are from Spain; a figure that grows every year, especially with the students of Asian origin, specifically from China.

In this research, we review the history of foreign language teaching in China, of Spanish teaching in China, and of Spanish teaching in SISU. Thus, the main objective of the research is to analyze the situation of the teaching of Spanish in China, showing how a language can become a cultural and language tourism resource for Spain.

### Spanish Teaching in China

As stated by the article, “Spanish in the World 2020,” published in the Spanish Yearbook 2020 (Instituto Cervantes, 2020), Spanish is the second mother tongue for a number of speakers after Mandarin Chinese, and the third language in the global count of users after English and Mandarin Chinese. On the Internet, Spanish is the third most used language: about 7.9% of users communicate in Spanish. It is the second language, next to English, in the publication of scientific texts. More than 9,07,000 foreigners come each year to study it, and they choose Spain for three main reasons: the cultural offer, the weather, and the attractiveness of the country.

The same book (Instituto Cervantes, 2020: 14–15) suggested that in 2020, there were more than 22 million students who learnt Spanish as a foreign language and in China, the figure was 55,285, which included 34,823 university students, 2,722 students in the IC in Beijing and Shanghai, and 8,866 studying in other institutions. As far as the total amount of the university students of Spanish is concerned, only the United States and Germany beat China with 7,12,240 and 52,947 apprentices.

Despite of those amazing data, Spanish teaching in China does not always enjoy a proper upsurge. In China, the total amount of apprentices of certain language seems to be crucial to classify a foreign language as a minor or a major language. After their hegemony of students' enrollment in the 1950s and in the 1980s, Russian and English have been considered as major languages, always considering the China's linguistic diversity (Ramsey, 1989). As a matter of fact, English, as *lingua franca*, has always been categorized as a major language, whereas other foreign languages are the minor ones. Based on the factors, such as development history, teaching scale, and employment market, the following languages are classified as major languages in this order: English, Japanese, Russian, German, and French (Lu, 2012). Until recently, Spanish had been considered as a minor language in China. Lu (2012) suggested that both minor and non-common languages are relative terms. As one of the six official languages of the United Nations, among Arabic, Chinese, English, French, Russian, and Spanish, Spanish has been picked out of the so called non-common languages group, losing preferential policies and extra support from the government during its foreign language education planning, especially after President Xi Jinping's Belt and Road Initiative. For example, the MOE of PRC published a list in 2010 announcing the establishment of National Training Base for Talent of Non-common language in eight universities, which means financial support and preferential recruitment and hiring of new faculty to non-common languages teaching departments. Shen (2019) suggested that since 2015, a “minor languages heat” has been coming into form with newly set majors or courses of languages spoken in Belt and Road countries in foreign language or comprehensive universities and that till 2019, about 47 professional teaching points for minor language majors were rapidly added to the countries. In most of the cases, teachers who have degrees in Spanish language in Chinese universities do not enjoy a preferential policy in performance evaluation in academia, which has been always biased toward research indicators. As Cadez et al. (2017) suggested that since “research-based performance evaluation in academia may be detrimental to teaching quality,” Spanish teaching in Chinese higher education institutions in China has to find a balance between teaching language skills and research tasks in linguistics, literature, and regional studies which might hold no interest for their students. The only solution seems to provide merely satisfactory teaching to guarantee more research time.

The political-economic relationship between China and the Hispanic world, especially Latin America, has been developing in the recent years, leading to an increase in the demand for people who speak Spanish in China, and to an increasing awareness of the meaning and value of the Spanish language as an asset and resource in the labor market (González-Puy, 2006; Lu et al., 2019). As a result, teaching and learning Spanish in China have undergone a remarkable boom in the past two decades. Currently, according to Zheng (2015); Zheng et al. (2019), there are more than fifty thousand students who have chosen Spanish as a foreign language in China. In addition, the report of the fifth symposium on “Teaching Spanish in China,” held in Beijing from July 15, 2018 to July 21, 2018, indicates that the number of Chinese universities with Spanish departments exceeds 90. It seems that there is a burgeoning number of Chinese people who are concerned about learning Spanish. According to the Yearbook of Instituto Cervantes (2021) and Ding et al. (2021), there are 101 universities in Mainland China with Spanish Department, where the language is taught as a bachelor's degree with a total enrollment of 25,000 students. The report of Lu et al. (2019) revealed that the spread of Spanish language and its culture, especially, the Latin American Spanish variant, is still quite limited compared to the expansion of English.

### Spanish Teaching in SISU

Diplomatic relations between China and Cuba were formally established in September 1960. Chinese People's Association for Friendship with Latin American and Caribbean Countries was founded in the same year. Lu (1992) mentioned the first boom of Spanish teaching in China around 1960 with the establishment of Spanish Department in the Shanghai Foreign Language Institute, the Shanghai Institute of Foreign Trade, the Peking University, the Beijing Broadcasting Institute, etc., as a result of increasing demand for talents with the knowledge of Spanish language and Spanish-speaking countries. In 1960s, Spanish courses were established in the Foreign Language School in Bejing and Shanghai and hundreds of young people entered the universities for a systematic education in the Spanish language and culture (Lu, 1992). A closer study on the history of Spanish teaching in SISU was made in the master thesis of Yang (2010), which was published in the digital journal of SinoELE, with the mention of Professor Xuhua Zhang, as the first teacher of Spanish in SISU in 1960. In the present article, we will focus on the teaching plan of SISU on Spanish, as a case study.

In accordance with the Discipline Catalogue of Degree Awarding and Talent Training promulgated by the MOE (Ministry of Education of PRC., 2021), in China, there are 13 discipline categories and 110 first-level disciplines. The 13 disciplines are as follows: 1. Philosophy 2. Economics, 3. Law, 4. Education, 5. Literature, 6. History, 7. Natural Science, 8. Engineering, 9. Agriculture, 10. Medicine, 11. Military Science, 12. Management Science, and 13. Arts.

Meanwhile the General Administration of Quality Supervision, Inspection and Quarantine and the Standardization Administration set another Classification and Code of Disciplines in China (GB/T 13745-2009) with 5 discipline categories and 58 first-level disciplines, according to which the 5 disciplines are as follows: 1. Natural Science, 2. Agricultural Science, 3. Medical Science, 4. Engineering and Technology Science, and 5. Humanism and Social Science.

**Figure 1:**
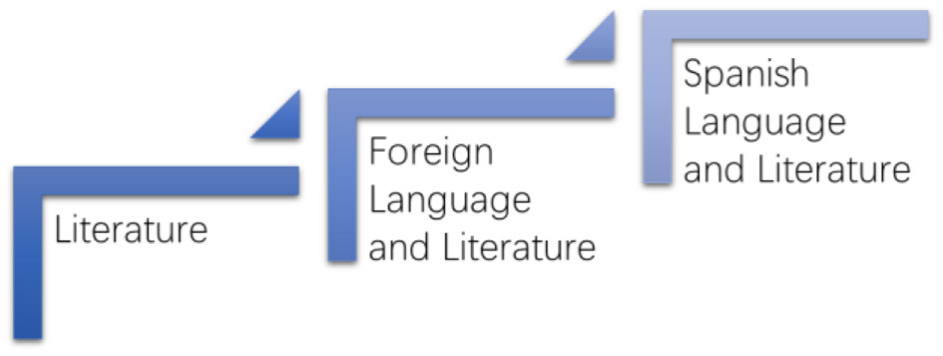
Classification of Ministry of Education (MOE). Source: Ministry of Education of PRC. http://www.moe.gov.cn/srcsite/A22/moe_833/201103/t20110308_116439.html.

**Figure 2:**
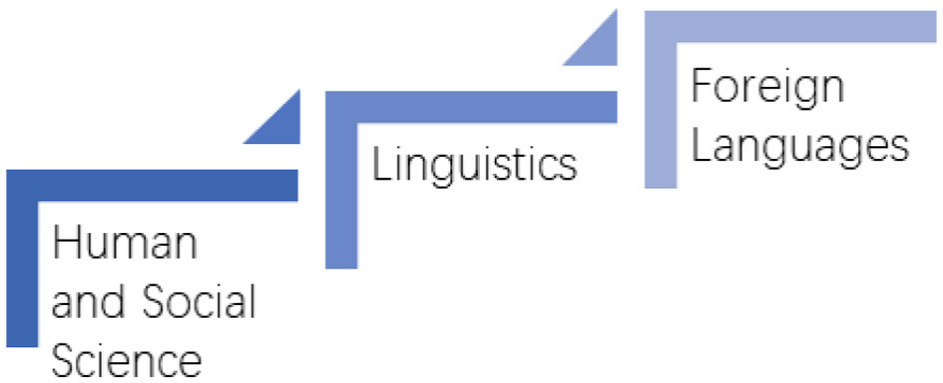
Classification of GB/T 13745-2009. Source: National Standard Full Text Open System. http://openstd.samr.gov.cn/bzgk/gb/newGbInfo?hcno=4C13F521FD6ECB6E5EC026FCD779986E.

Therefore, two tables can be drawn according to the two classification standards (Figures 1, 2).

In Spain, the specific phase of the test will be structured in five options linked to the five branches of knowledge established by Real Decreto 412/2014 (Agencia Estatal Boletín Oficial del Estado (B.O.E, 2014). https://www.boe.es/eli/es/rd/2014/06/06/412:) option A (arts and humanities); option B (natural sciences); option C (health sciences); option D (social and legal sciences); and option E (engineering and architecture). Evidently, the Spanish Language and Literature in Spain, which belongs to Arts and Humanities, has different intensions or extensions. As we can observe in the website of Universidad de Alcalá (UAH) and Universidad Autónoma de Madrid, Spanish studies are included in the degree programs of their School of Philosophy and Literature (Facultad de Filosofía y Letras) (Universidad de Alcalá., 2021; Universidad Autónoma de Madrid, 2021)^1^,^2^.

In China, taking the BFSU and the SISU as examples, the Spanish studies is incorporated, respectively, in the School of Hispanic and Portuguese Studies and the School of European and Latin American Studies. In the School of European and Latin American Studies of SISU, there are 5 degree-programs, such as Spanish, Portuguese, Italian, Greek, and Dutch studies, whereas in BFSU, 2 programs of degree study, Spanish and Portuguese studies, form an independent school. These two typical “foreign language studies” universities still maintain the tradition of dividing its schools according to the languages instead of the discipline criteria. In the same Spanish major, there might be different directions, such as translation, literature, linguistics, and regional studies. According to the teaching plan of SISU, the bachelor's degree in Spanish consists of 160 credits, where 1 credit point takes 18 h of lecture (1 lecture hour = 45 min), that is 13.5 h without counting the extra hours outside of the classroom. In UAH, Hispanic Studies require 240 European Credit Transfer System (ECTS), where a 6-ECTS-point course takes about 48 h of class and 102 h for students' own work, that is to say, 1 ECTS credit point takes 8 h of lecture plus 17 h on students' own time. In the common practice of credit transfer, 1 Chinese credit equals to 1.5 ECTS credits. During 160-credit-point Spanish Major in SISU, 88 credits are dedicated to the Spanish language and culture learning, whereas in UAH, most of subjects are related to Spanish linguistics and literature (Table 1).

**Table 1:** Spanish-related subjects in the teaching plan of SISU.

**Type of subject**	**Course**	**Credit(s)**	**Lecture hours**
Basic subjects	Basic Spanish I/II	32	648
	Spanish Reading I/II	6	144
	Spanish Audiovisual I/II	6	144
	Advanced Spanish I/II	16	288
	Spanish Writing I/II	2	36
	Translation Theory and Practice (Spanish to Chinese)	4	72
	Translation Theory and Practice (Chinese to Spanish)	4	72
Compulsory subjects	Overview of Spain	2	36
	Overview of Latin America	2	36
	Literature of Spain	2	36
	Literature of Latin America	2	36
Optional subjects I	Spanish Phonetic	1	18
	Spanish Conversation	2	36
	Spanish Grammar	2	36
	Spanish Interpretation	1	18
	Selected Readings in Spanish	2	36
	Spanish Listening	2	36
	Spanish Correspondence Writing	2	36
Optional subjects II	Administration Management (Spanish)	2	36
	History of Spain	2	36
	Spanish Morphology	1	18
	Spanish Lectures or Conferences	4	72
	Spanish Rhetoric	1	18
	Brief History of Relationship between China and Latin America	1	18
	Commercial Writing in Spanish	2	36
	History of Latin America	2	36
	Foreign Related Secretary	2	36

*Source: Retrieved from the website of SISU. http://www.newoaa.shisu.edu.cn/_upload/article/files/ce/83/cf352b0e4c839c8ade79f06f590e/de383f3e-943e-49d7-9ec1-076da1210703.pdf*.

**Figure 3:**
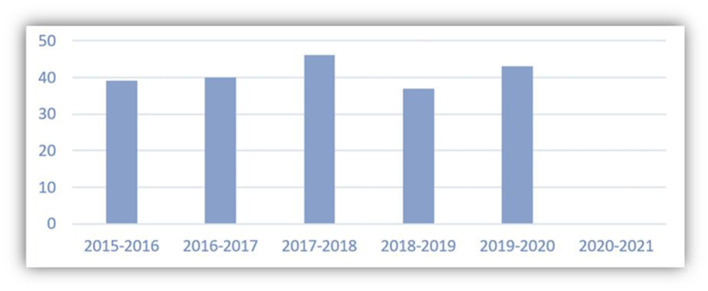
Number of exchange students of SISU in the Spanish speaking countries (Bachelor of Arts). Source: Own elaboration with data from the Office of International Affairs of the School of European and Latin American Studies of SISU.

The Shanghai International Studies University has an enrollment of about 60 students each year in a 4-year program of Hispanic Studies (Figure 3). There are around 240 graduate students of Spanish in the University. Study Abroad programs often occur in the fifth, sixth, and seventh semesters, which can last one semester or the entire academic year depending on the will of each student and the cooperation agreement with each foreign institution. From 2015 to 2020, there were 205 graduate students who participated in the Study Abroad programs. Due to the global pandemic, SISU has suspended all its international exchange programs during the 2020–2021 academic year, which explains the 0 number in the last column of Table 6. Among these 205 exchange students, 156 chose to study in Spain, which represents 76% of the total Study Abroad participants. About 24% of the total students chose to study in Mexico, Chile, and Argentina. Among the 156 students in Spain, 124 have chosen to study in Madrid, which accounts for 79.5% of the total exchange students of Spanish in SISU, as shown in Table 7. Also, we observe that UAH occupies the first place in accepting a majority of students (77) from SISU, which benefits from internationalization strategies of SISU, specially of the School of European and Latin American Studies for Hispanic Studies. Basically, three factors have been taken into consideration while planning an international strategy. First, it is important to determine the strategic countries because it would be impossible to try to establish a cooperation with all the Spanish-speaking countries. Considering the linguistic variety and cultural diversity, Spain and Mexico remain as our strategic countries for international exchange and cooperation for Hispanic Studies. With social and economic peculiarities of Chile and Argentina, these two countries also arouse great interest in the academic cooperation. It is worth mentioning that transportation convenience and visa policies determine the favorite destinations for the Study Abroad programs for the students. For example, the government of Spain optimizes the student visa procedure, while Spanish universities carry out preferential policies to attract the Chinese students. Beltran (2011, 90) suggested that “The attempts made by Spanish universities to attract future students from China are increasingly successful. During the last years, it has been shown that far from coming to Spain with the intention of staying, the vast majority do so to get a degree and an international experience that, in addition to opening new fields in their professional future, at the time of their return to China compensates them for the expectation of obtaining better salaries compared to those who have not studied abroad.”

**Figure 4:**
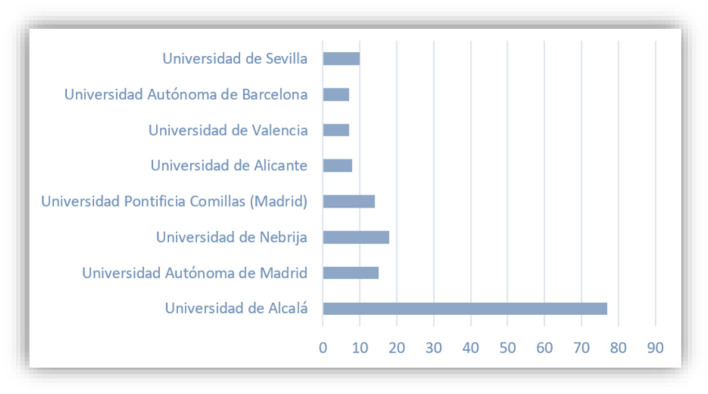
Spanish universities that accept 156 SISU students since 2015. Source: Office of International Affairs of the School of European and Latin American Studies of SISU.

Second, it is important to maintain those strategic partnerships as truly active. In this case, the UAH is the target university with a corresponding teaching quality, research output, and international outreach. Third, it is always an effective way to keep active exchange and cooperation by implementing key projects and programs. The UAH is the first Spanish university that signed a cooperation agreement with SISU. Additionally, in 2014, the two universities have successfully applied a joint program as a pilot project of SISU on Chinese-Foreign Cooperation in Running Schools approved by the MOE of PRC. Both sides agreed on that during the fourth, fifth, sixth, and seventh semesters, a Spanish faculty from the School of Economics and Business Sciences and Tourism (Facultad de Ciencias Económicas, Empresariales y Turismo) come to SISU and give in-person classes of 8 subjects on Economics and Business Administration. Those three factors led to the fact that UAH received a majority of SISU students in Spain, as demonstrated in Figure 4.

### Spain: A Destination for Language Tourism in China

In 2001, Turespaña proposed a series of actions to strengthen the tourism sector in the country and, thus, deseasonalize tourism, among other things. In this context, the historical, artistic, and linguistic heritage of Spain was a fundamental element to be developed. At the same time, the teaching of Spanish as a tourist resource showed numerous shortcomings. Thus, in 2001, the Plan to Promote Cultural and Language Tourism was born (Güemes, 2001). This plan reflected the strengths and weaknesses of the language and cultural offer in Spain and proposed six axes of action through 40 measures. Specifically, in the fifth axis, the teaching of Spanish as a tourist resource had an independent consideration due to its specific nature of both supply and demand. In it, the concept of displacement to Spain was presented with the motive of learning the Spanish language as a by-product of cultural tourism and it also had a greater attraction for Spanish tourism in general, both for the direct or indirect economic repercussions and the contribution of the image of destination. This plan also includes measures related to the instruments of promotion and commercialization of the teaching of Spanish as a tourist resource.

Turespaña, recognizing that the segment presented a potential growth and consequently a form of diversification of the tourist offer in Spain, published two reports within the series of Studies of Tourism Products, the first in March 2001 entitled, “Language Tourism” and later, “Language Tourism” in October 2008. While it is true that since that date, these reports have not been published with continuity; these studies serve to show the potential markets and the characteristics of the language tourists who arrive in our country and redirect specific policies of commercialization of the product.

According to the data provided by the IC, the annual number of language tourists is estimated to be 2,000,000, of whom 2,73,000 are from Spain, a figure that grows every year.

The data collected by FEDELE in its study, “Spanish in Spain, a sectoral report” (2018) recorded the following distribution according to the origin of visitors: about 80.3% come from Europe, 7.7% from America, 9.3% from Asia, 1.1% from South America, and 0.8% from Africa. The IC, through the information reported by accredited centers, ensures that in the recent years, there has been a very significant increase in the students of Asian origin, especially from China.

Spain is a mature tourist destination, second in the reception of tourists from all over the world. It currently aims to diversify its tourism offer in order to reach potential consumers as growth strategies. Chinese tourism is becoming one of the most important targets for destination offers, mainly in Europe.

Turespaña is working on specific strategies for the attraction of the Chinese market to Spain, especially based on the offer of cultural tourism packages. Language tourism is currently growing and its characteristics arouse an important interest for cities because of its impact.

According to Taboada de Zúñiga (2010), the promotion policies of this new sector in Spain have had different drivers at the state, regional, and local levels: Turespaña, FEDELE, the IC, the Ministry of Culture, the Spanish Institute of Foreign Trade (ICEX), the Spanish Agency for International Development Cooperation (AECID), the EDUESPAÑA association, and the autonomous communities and local entities.

The Chinese market is an emerging market that interests Turespaña because it is a substantial market, with economic capacity and with an interest in culture, an essential tourist product for the tourist diversification of the Spanish tourist market very dependent on the “sun and beach” tourism (Turespaña, 2008, 2021).

As of 2014, China overtook the United States as the world's largest overseas travel market, both in terms of arrivals generated and total travel expenses. If the purchase intention of Chinese tourists is analyzed, the first variable that influences their purchase decision process is the visit to the countries near China, both geographically and culturally. These countries are South Korea, Japan and Southeast Asia. Next, Australia and New Zealand are mentioned, which are relatively closer destinations, well connected with direct flights to China and with a strong tourism promotion strategy. As for our European competitors, the United Kingdom and France are positioned ahead of Spain (According to the 2017 report prepared by the China Tourism Academy (CTA).

The main strengths of Spain for the Chinese market (García-Henche and Qi, 2019) are the richness and diversity of cultural, urban, and gastronomic offer and diversified portfolio of attractive thematic tours for the Chinese, the highly developed tourism infrastructure, security, and excellent health services, stable bilateral relations, Chinese capital investments in Spain, and the perception of Spaniards as hospitable and open to the reception of Chinese tourists.

These strengths generate business opportunities due to the increase in flight connections, a consolidated senior Chinese tourism, the enormous potential of convention and congress tourism in the Chinese outbound market, the forecasts that China will double its spending on international tourism compared to 2016 (450.00 million USD), and the increase in the demand for studies in Europe.

Taking advantage of this last aspect, the IC and the Education Council work to reach Chinese students through different communication tools, especially Chinese social networks, such as Weibo [Sina Weibo (Chinese: 新浪微博, pinyin: Xinlang Weibo), is a Chinese social media website, similar to Facebook and, to a lesser extent, Twitter. It is used by ~30% of Internet users in China] and WeChat (WeChat in Chinese: 微信, pinyin: Weixin, literally “micro-message.” It is a mobile text messaging service and voice message communication service created by Tencent, China factory, launched in January 2011).

Turespaña work on improving the presence in these social networks, since it analyzes the presence of the brand, Spain as a tourist destination. It can be said that to penetrate the Chinese tourist market, it is necessary to strengthen the presence of Spain in the most important Chinese Social Networks and digital media (especially, WeChat and Weibo) for the decisive influence they have among the millennial generation (Generation Y), who are a new segment of international tourists of great importance on the following factors: their purchasing power, their greater command of languages, which allows them to travel independently without being captive to the traditional European markets of tour operators, and for the interest they have in Europe. This generation discovers, plans, and buys their trips largely through the Smartphone and maintains a very active presence on the Chinese social networks.

Thus, both Turespaña and the IC work on positioning in these communication networks.

Similarly, from the different universities that work with Spanish studies, as is the case of SISU, it is sought to generate the image of the studies of Spanish as a language and of the universities with which collaboration agreements are made. This is perceived on the website of the University of SISU, where the approval of the Chinese MOE on the agreement of the UAH and SISU for studies on Spanish and Business Sciences is announced.

The information of communication in networks regarding Spanish as a cultural resource in China, shows how language can become a culture and language tourism resource for our country. Thus, the Spanish language grows as an economic resource because the economic and consumption capacity of its community of speakers, both Spanish speakers and Spanish learners, grows. This means that the Spanish language is studied and learned by thousands and thousands of foreigners, particularly from Europe, the United States and China, our object of study.

In addition, education is one of the focuses of attention for SDGs. The SDGs are calls for action by all countries—poor, rich, and middle-income—to promote prosperity while protecting the planet. They recognize that ending poverty must go hand-in-hand with strategies that build economic growth and address a range of social needs including education, health, social protection, and job opportunities, while tackling climate change and environmental protection.

Thus, education is an important value for SDGs, and therefore, it could be said that Spanish has become a resource for the Chinese market to develop quality education and international cooperation, as proposed by the fourth and seventeenth SDGs (Sustainable Development Goals, 2021.

## Methods and Survey Design

This research is based on a descriptive study using primary data from a questionnaire used on a representative sample of students of Spanish degree study. A questionnaire made of 11 questions was published on an open survey platform, Wenjuanxing (Wenjuanxing, in Chinese, 卷星, which means Star of Questionnaire, with official website www.wjx.cn, is a widely accepted online questionnaire survey platform in China for data collection) and a total of 159 students accessed the online survey and completed the questionnaire from April 7, 2021 to May 1, 2021.

In addition, the secondary data of the websites of the IC and TurEspaña in the Chinese market and the use of social networks as an instrument of communication with that market have been reviewed, to present an idea of the brand image that Spain sells in China as a tourist destination.

## Results

**Figure 5:**
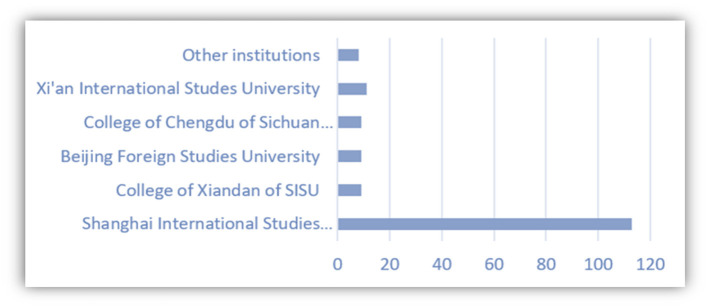
Institutions of respondents.

**Figure 6:**
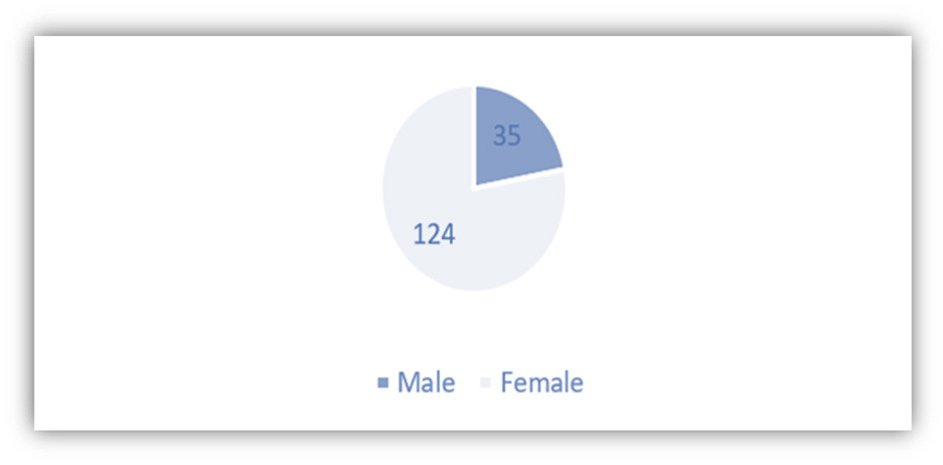
Gender distribution of the respondents.

The purpose of analyzing the information collected is to know relevant information on the profiles of Spanish students in China. About 117 out of 186 undergraduates of the Spanish Department of School of European and Latin American Studies of SISU have participated in the survey (refer to Figure 5). One of the main characters of the students of Spanish, or foreign language students in the Chinese universities that has been reflected also in Figure 6, after the survey, is that there are more female students than male students in the foreign language schools or universities.

This study aims to publicize the situation of teaching of Spanish in China, showing how language can become a culture and language tourism resource for Spain.

Consumers interested in this kind of linguistic tourism, which essentially includes language and culture, belong to a greater variety of social classes and types. We can find young people interested in traveling, working, or studying, people with business interests, and people who seek cultural experiences, interested in combining language learning and other types of activities not necessarily touristic in a traditional sense but within a perspective of intercultural development—this includes retired people who have time and means which allow them to travel and enjoy new linguistic and cultural experiences (Baralo, 2015). Linguistic tourism or language study stay (language visit) generates situations which constitute an enriching experience not only for those who travel but also for those who receive them in their countries.

The touristic policies of Spanish-speaking countries should aim at occupying a central position in the world educational economy. In order to achieve it, it is essential to create products and services with greater added value, promote the convergence of suppliers of educational services and product editors, stimulate the demand of Spanish in new markets, and improve the adaptation of educational products and services to specific markets, with a special attention to the use of the internet and learning and communication technologies.

**Figure 7:**
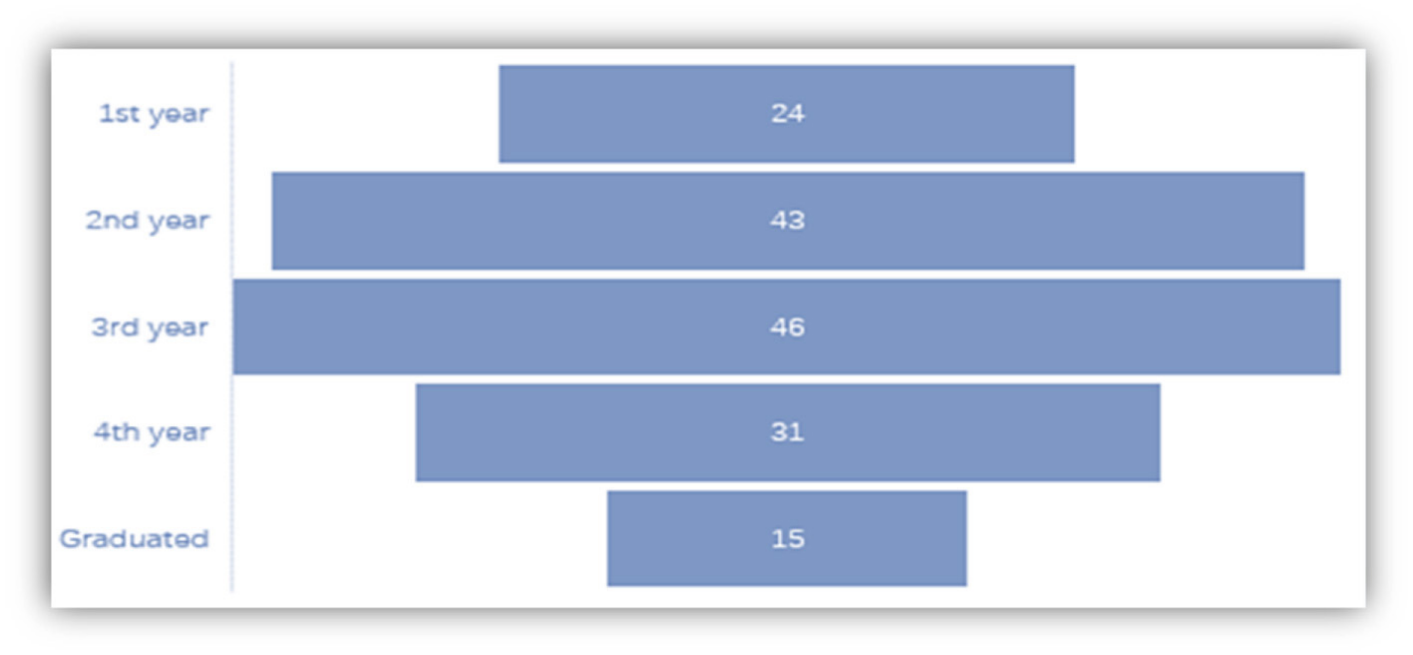
Grade distribution.

As it was expected, most of the respondents are in sophomore and junior years in the university, when students are encouraged to participate in the Study Abroad programs (Figure 7).

**Figure 8:**
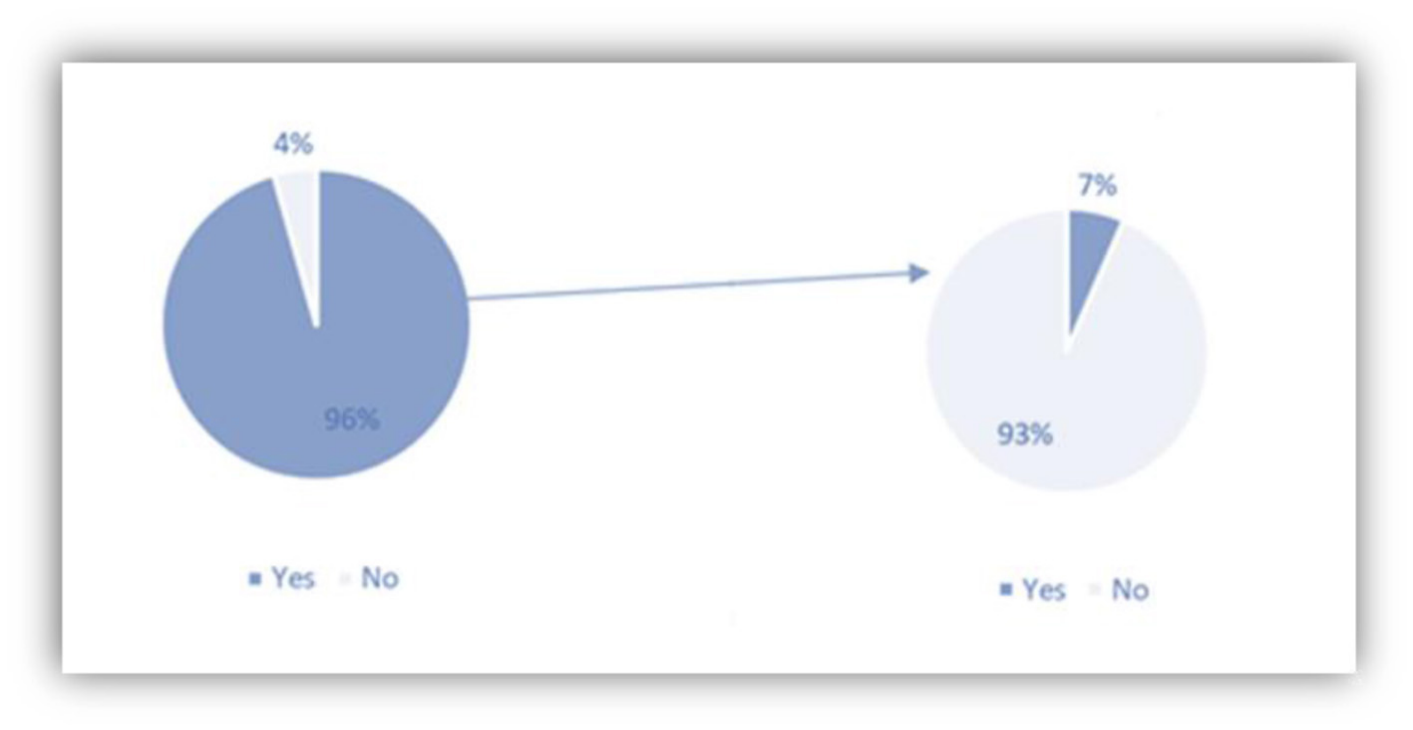
Knowledge of the IC and learning experience in IC.

As explained above, the IC is responsible for the promotion and training of Spanish as a language resource in the world. The Instituto Cervantes, 2006 was created in Spain in 1991 to promote the Spanish language and cultures of Spanish-speaking countries. The IC in Beijing was founded on 14th of July 2006^3^ A year later, the Biblioteca Miguel de Cervantes was founded in Shanghai as a public institution attached to the Consulate General of Spain in Shanghai and IC. About 96% of our respondents have heard of IC. Among these 152 students, only 7% of them have taken some Spanish course with IC. Judged by the cultural activities organized by IC in Beijing or Biblioteca Cervantes in Shanghai, their target clients or students are not undergraduates from universities but the so-called, “social people,” which is a concept that excludes students from higher education institutions and might refer to Spanish language and culture lovers from all walks of life. Additionally, fewer cultural activities of IC in collaboration with SISU also prove this hypothesis. On the other hand, students of Hispanic Studies in the Chinese universities might think that those cultural activities and language courses are more suitable for beginners instead of undergraduates. Actually, more communications are required to promote synergistic cooperation among IC and Spanish teaching universities (Figure 8).

**Figure 9:**
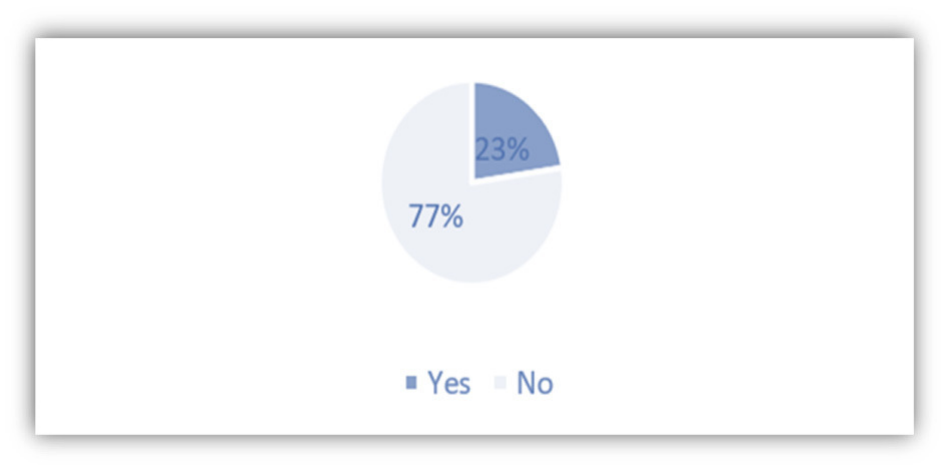
Study experience in Spain.

An important fact to know how the language can become a tourist resource is to know if students visit Spain to carry out studies related to the language. As COVID-19 has jeopardized severely the Study Abroad programs of universities during the 2020–2021 academic year, including the SISU, there are 77% of students that have not yet had any study abroad experience in Spain. On the other hand, about 23% of students have been studying in Spain (Figure 9).

**Figure 10:**
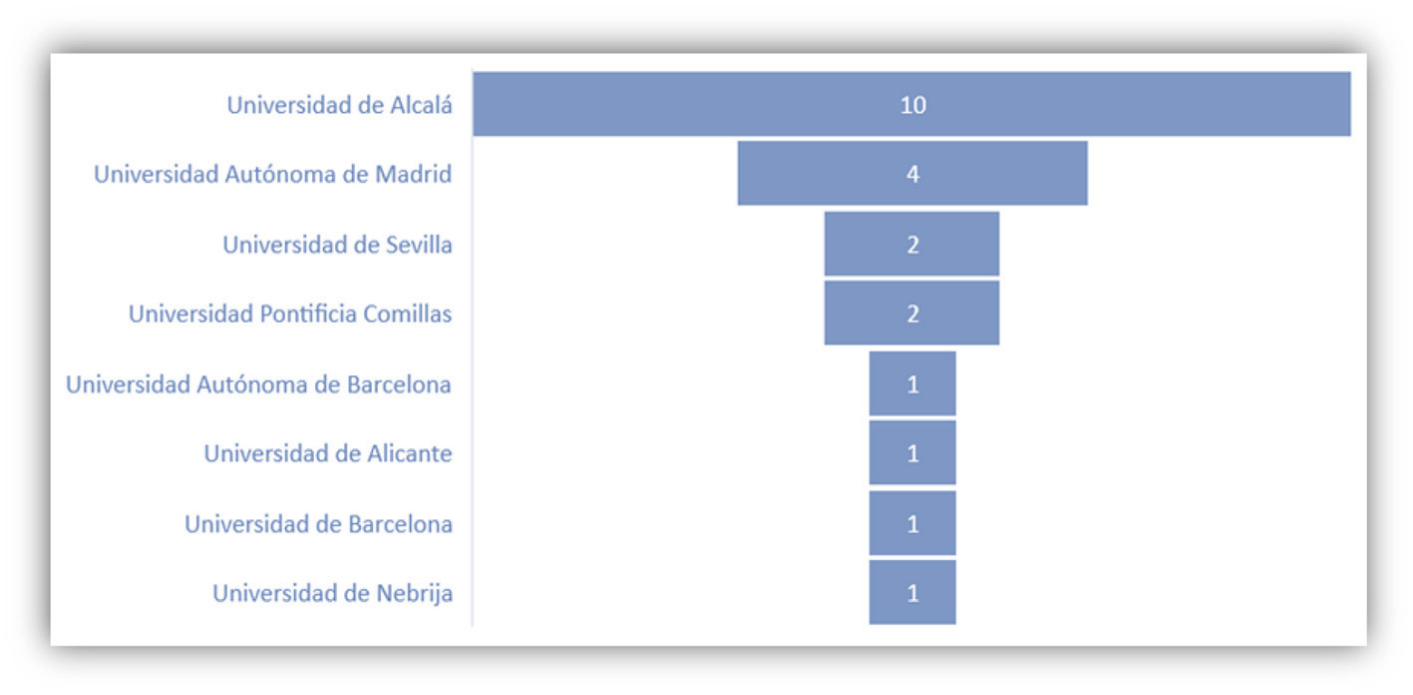
Most visited Spanish universities.

**Figure 11:**
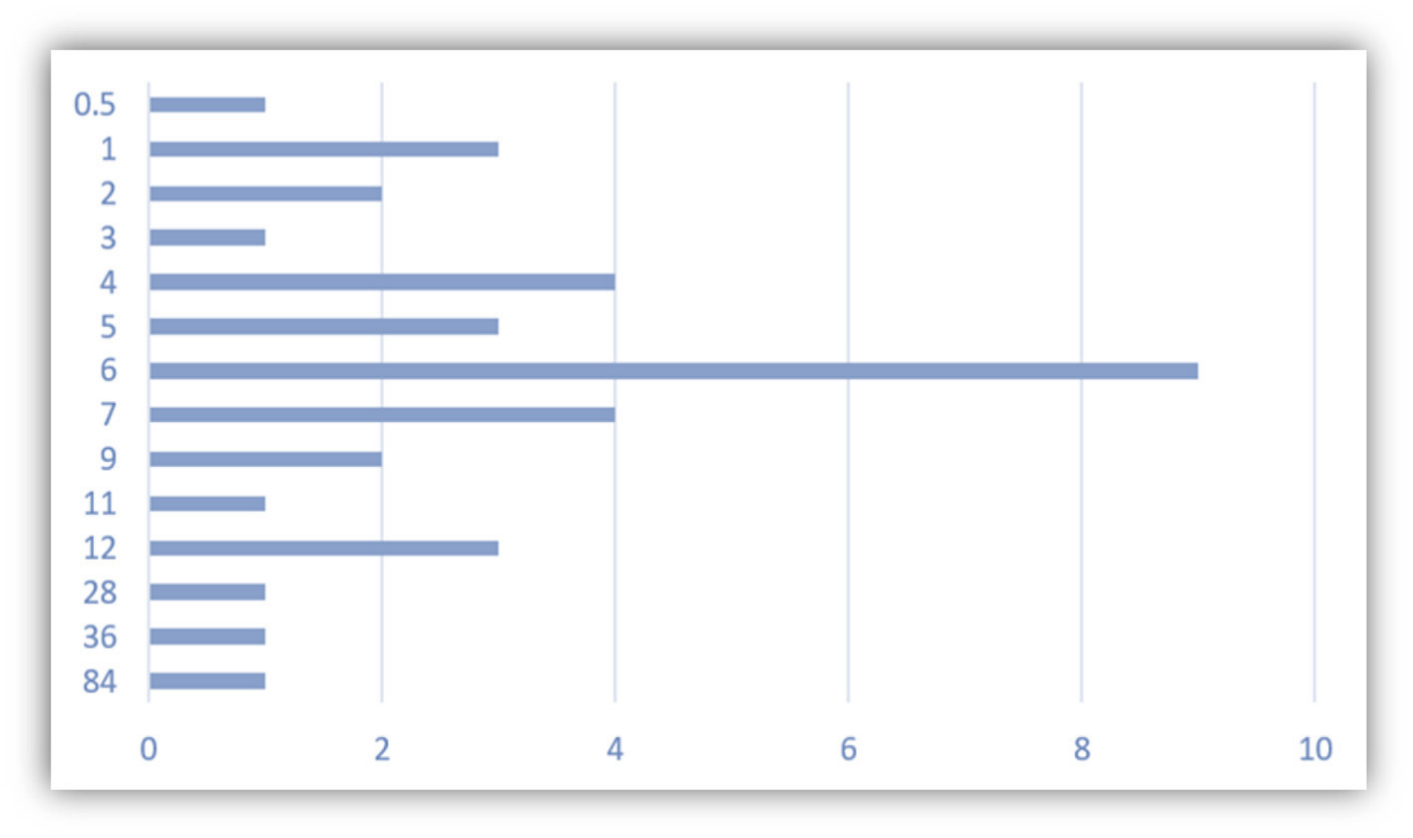
Number of months of study in Spain.

In Figure 10, we can find most visited Spanish universities as one of the results of this survey. The SISU has cooperation agreements with nearly all of them, except the Universidad de Barcelona. In addition, among the 36 students who have studied in Spain, most students chose the 6-month Study Abroad programs, with some exceptional cases in which students have stayed more than 1 year in the country (Figure 11).

**Figure 12:**
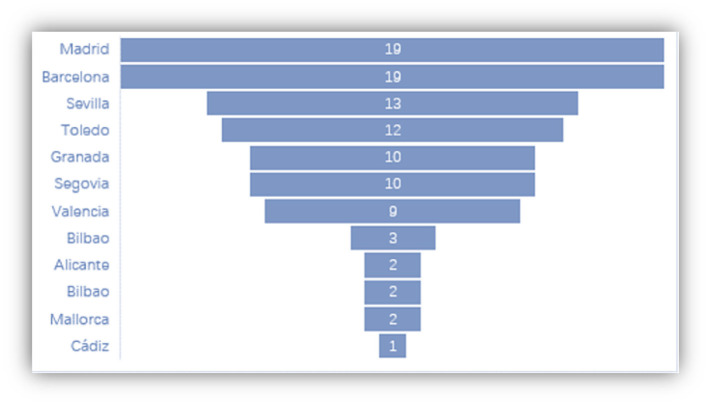
Most visited Spanish cities by the Chinese students.

**Figure 13:**
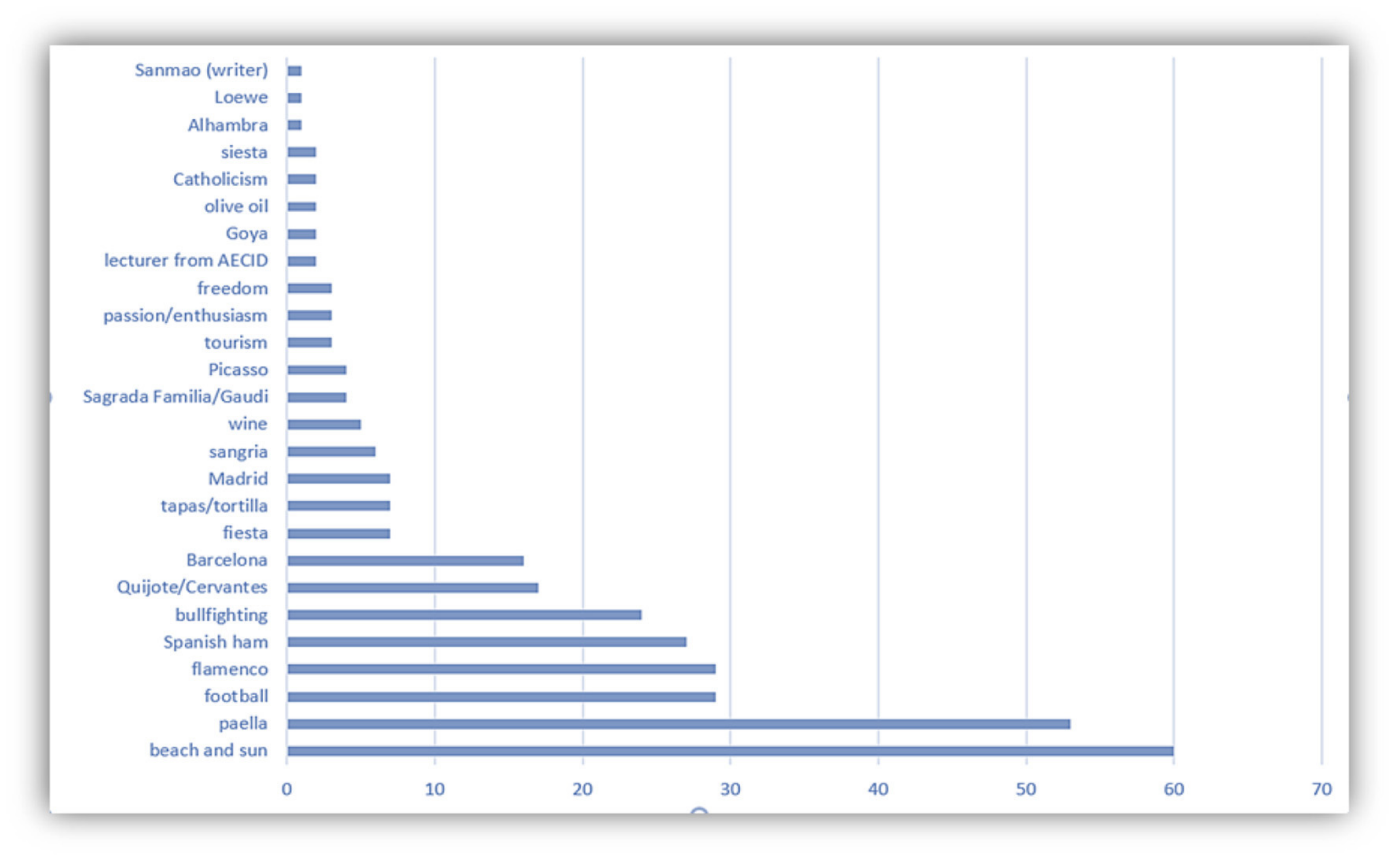
Most mentioned words related to Spain.

Part of the questions in the questionnaire are associated with variables related to tourism, such as the most visited cities or the image of Spain as a tourist resource (Figures 12, 13). The last question in the survey shows some closely related words to Spain. As the ranking shown in Figure 13, even for students of Hispanic Studies, many stereotypes about this country are on the list, like flamenco, bullfighting, nap-taking, paella, sangria, Madrid and Barcelona, and fiesta. But it would be a mistake to put an equal sign between stereotype and misconception. Although not all Spaniards live in Madrid and Barcelona, these two metropolitan cities are definitely a showcase of cultural attractions. But at the same time, students who know better Spanish language and culture would like to consume some tourism package created for niche market. For example, in Gran Canaria, “la Ruta Sanmao,” a niche-market target tourism package has been designed to attract those fans of Sanmao, a famous Chinese writer who lived for many years on the island with her Spanish husband, José María Quero.

## Discussion and Conclusions

### Theorical Discussions

This study aims to analyze how language tourism and cooperation between cultural and tourism institutions and universities, in line with the SDG numbers, 4 and 17 of the World Tourism Organization (UNWTO), 2021, can serve to sustainably develop quality education (SDG4) and international cooperation (SDG17).

The rapid increase in disposable per capita income in China makes the overseas travel increasingly accessible to an increasing number of consumers. Chinese outbound tourism is not only growing in size but also maturing and becoming more diverse. The fact that more and more Chinese citizens want to travel independently means a change in the offer of tour packages for personalized trips. Spanish tourism companies can also benefit from this market demand. In doing so, they must ensure that their offers are designed to accommodate the demand of their Chinese customers.

Due to the increase and interest in the Spanish language in China, especially at the University level, a new market niche is generated for language tourism. Therefore, it would be advisable to personalize the trips and ensure that the information is available in Chinese in its search engines and social networks for this market niche.

This market of Chinese students has a concept of Spain associated with cultural values, such as literature, gastronomy, and cultural patrol that are associated with the studies they carry out. Therefore, looking at the future, if incomes and educational levels continue to grow, Chinese outbound tourism will not only increase but will also be more sophisticated and demanding as is the case with language tourism.

Destinations that offer cultural and urban tourism, especially destinations with prestigious universities, will have a business opportunity in this new Chinese market, if they know how to meet the needs of these tourists and offer information to prepare the trip and tourist products adapted to the new profiles of consumers.

### Managerial Discussions

From a managerial point of view, this research can assist all those authorities who have an influence on educational and linguistic policies in Spain.

The Spanish language is considered internationally as the second mother tongue, which generates an increase in foreign tourists in order to learn the language and get to know our culture. For this reason, it can be considered a typology of emerging tourism that copes with the seasonality of demand, distributing it practically homogeneous throughout the year. This produces an increase in the number of Spanish centers for foreigners and, in addition, more and more schools are working to obtain accreditation from IC.

Castillo et al. (2018) highlighted in their study, the importance of the role of the international university student as an engine for the promotion of the development of international educational tourism as a modality within nautical tourism. They highlighted the importance of international educational tourism to promote the diversification of the tourism product of a country and, in this way, contribute to its economic growth.

One of the main weaknesses regarding language tourism is the lack of actualized studies carried out by the administration, so it would be interesting for the institutions responsible in the different public administrations of the Spanish-speaking countries to jointly prepare a quantitative and qualitative study of the current state and future possibilities of language tourism, specifically in the Chinese market since it is one of the fastest growing markets, according to the different studies of language academies.

It is necessary to collect more data on language tourism, which has few official figures. It is important to measure the economic impact of this sector in the different regions and cities, deepening the expenditure and the distribution of it. Above all, this type of tourism is a product of great value, for its profitability, and for its contribution to territorial deconcentration and deseasonalization. The students of today are the tourists of tomorrow and, for example, the facility that social networks grant today, which they usually handle, make them great diffusers of the Spain brand.

Modern management experts believe that coordination is the essence of management (Koontz et al., 1986). In SDGs-oriented management, coordination is the core link, as the purpose of coordination is not only to integrate the various components, but also to synchronize the functions of the various departments to achieve the SDGs with minimal effort. In doing so, first, we should strengthen the coordination between the SDGs. This means that we must analyze the interactive mechanisms between each goal based on the classification discussed above and should involve a consensus of the multidisciplinary scientific community.

Inter-university collaboration is an example of the orientation of SDGS, when universities work with cooperation programs, such as ERASMUS Program or Erasmus+ KA107 which provide opportunities for students to complete their academic training and university staff to exercise teaching functions or train in higher education institutions outside Europe and vice versa, or the numerous cooperation programs between Spanish and Chinese universities (for example, the UAH has cooperation agreements with various Chinese universities, such as SISU, the Jinan University, the Beijing Institute of Technology, and the Dalian University of Foreign Languages) and even between institutions, such as the cultural and linguistic cooperation agreement between the RAE and the SISU.

It would be advisable to have a careful analysis that contemplates all the agents involved, both public and private, and both state, regional, and local, in the promotion of this tourism, with the intention of assessing the work that is being done and proposing ways of coordination and collaboration to promote, improve its quality, and increase its profitability, especially in the Asian market niches, as is the case in China.

### Limitations and Future Research

The main limitation of this study is that we have focused on a university group of students. It would be convenient to apply this methodology to a complete study, focusing on the most popular universities in China.

Another future line of research would be to extend the study to other different countries to obtain different conclusions about the linguistic behavior of the students, not only to study in Spain but also in the Latin American countries.

## Conclusion

This study is important to be able to make decisions, especially from the point of view of obtaining the development of quality education (SDG4) and international cooperation (SDG17) as SDGs. It has analyzed language tourism, taking into account the three dimensions of sustainable development: economic, environmental, and social.

Tourism includes language as a cultural resource for the temporary journey to some destinies. This kind of tourism is still not very known in our country, and this article is one of the first contributions in the field of education and tourism. Designated as language tourism, this investigation focuses on Spanish Studies in China as a market segment that represents an opportunity for language tourism in Spain.

The results demonstrate that language tourism has relatively a greater economic impact than conventional tourism, that its negative effects on the environment are not significant; it is beneficial for future generations, since it contributes to the increase in knowledge and interrelationships among students of different countries and cultures.

## Data Availability Statement

The raw data supporting the conclusions of this article will be made available by the authors without undue reservation.

## Author Contributions

BG-H and MY: conceptualization, methodology, software, validation, formal analysis, investigation, resources, data curation, writing—original draft preparation, and writing—review and editing. All authors have read and agreed to the published version of the manuscript.

## Conflict of Interest

The authors declare that the research was conducted in the absence of any commercial or financial relationships that could be construed as a potential conflict of interest.

## Publisher's Note

All claims expressed in this article are solely those of the authors and do not necessarily represent those of their affiliated organizations, or those of the publisher, the editors and the reviewers. Any product that may be evaluated in this article, or claim that may be made by its manufacturer, is not guaranteed or endorsed by the publisher.

## Footnotes

^1^ https://filosofiayletras.uah.es/estudios/grados.asp

^2^ http://www.uam.es/FyL/Grado/1234889902868.htm?language=es&nodepath=Grados%20y%20Dobles%20Grados&pid=1234889900523

^3^Information retrieved from website of Instituto Cervantes de Pekin https://pekin.cervantes.es/cn/about_us_centre_spanisl.htm.
